# Relacorilant plus nab-paclitaxel for the treatment of metastatic pancreatic ductal adenocarcinoma: results from the open-label RELIANT study

**DOI:** 10.1093/oncolo/oyae210

**Published:** 2024-08-26

**Authors:** Erkut H Borazanci, Nathan Bahary, Vincent Chung, Timothy K Huyck, Ebenezer A Kio, Elena Gabriela Chiorean, Roland T Skeel, Olatunji B Alese, Dana B Cardin, Christos Fountzilas, Wahid T Hanna, Alexis D Leal, Valerie Lee, Anne M Noonan, Philip A Philip, Zev A Wainberg, Hristina Pashova, Grace Mann, Paul E Oberstein

**Affiliations:** HonorHealth Research Institute, Scottsdale, AZ, United States; Allegheny Health Network Cancer Institute, Pittsburgh, PA, United States; City of Hope Comprehensive Cancer Center, Duarte, CA, United States; Nebraska Cancer Specialists, Omaha, NE, United States; Goshen Center for Cancer Care, Goshen, IN, United States; University of Washington, Fred Hutchinson Cancer Center, Seattle, WA, United States; University of Toledo Medical Center, Toledo, OH, United States; Winship Cancer Institute, Emory University, Atlanta, GA, United States; Vanderbilt-Ingram Cancer Center, Nashville, TN, United States; Roswell Park Comprehensive Cancer Center, Buffalo, NY, United States; University of Tennessee Graduate School of Medicine, Knoxville, TN, United States; University of Colorado School of Medicine, Aurora, CO, United States; Johns Hopkins University School of Medicine, Baltimore, MA, United States; The Ohio State University, Columbus, OH, United States; Karmanos Cancer Institute, Wayne State University School of Medicine, Detroit, MI, United States; University of California School of Medicine, Los Angeles, CA, United States; Corcept Therapeutics Incorporated, Menlo Park, CA, United States; Corcept Therapeutics Incorporated, Menlo Park, CA, United States; New York University Langone Health, New York, NY, United States

**Keywords:** nab-paclitaxel, metastatic pancreatic cancer, PDAC, relacorilant

## Abstract

**Background:**

Modulation of glucocorticoid receptor (GR) activity in tumor cells enhances chemotherapy efficacy. We evaluated the selective GR modulator relacorilant plus nab-paclitaxel in patients with metastatic pancreatic ductal adenocarcinoma (mPDAC) who had received at least 2 prior therapy lines.

**Patients and Methods:**

In this open-label, single-arm, phase III study, patients received once-daily oral relacorilant (100 mg, titrated to 150 mg in 25 mg increments/cycle) and nab-paclitaxel (80 mg/m^2^) on days 1, 8, and 15 of 28-day cycles. The primary efficacy endpoint was objective response rate (ORR) by blinded independent central review. Progression-free survival (PFS), overall survival (OS), target gene modulation, and safety were also assessed.

**Results:**

Of 43 patients enrolled, 31 were evaluable for ORR (12 did not reach first postbaseline radiographic assessment). An interim analysis to assess whether ORR was ≥10% showed no confirmed responses and the study was discontinued. Two (6.5%) patients attained unconfirmed partial responses and 15 (48.4%) had stable disease. Fourteen of 31 (45.2%) patients had reductions in target lesion size, despite prior nab-paclitaxel exposure in 12 of the 14. Median PFS and OS were 2.4 months (95% CI, 1.4-4.2) and 3.9 months (95% CI, 2.8-4.9), respectively. The most common adverse events were fatigue and nausea. RNA analysis confirmed that relacorilant plus nab-paclitaxel suppressed 8 cortisol target genes of interest.

**Conclusion:**

Relacorilant plus nab-paclitaxel showed modest antitumor activity in heavily pretreated patients with mPDAC, with no new safety signals. Studies of this combination in other indications with a high unmet medical need are ongoing.

## Teaser

Modulation of glucocorticoid receptor (GR) activity in tumor cells may restore chemosensitivity and enhance chemotherapy efficacy. This study evaluated the selective GR modulator relacorilant plus nab-paclitaxel in patients with metastatic pancreatic ductal adenocarcinoma who had received at least 2 prior therapy lines.

**Implications for Practice**

We evaluated nab-paclitaxel plus the selective glucocorticoid receptor (GR) modulator relacorilant—a therapy that may enhance chemotherapy sensitivity and efficacy—to treat patients with metastatic pancreatic ductal adenocarcinoma (mPDAC) who had received ≥2 prior therapy lines. Of 31 efficacy-evaluable patients, 2 (6.5%) attained unconfirmed partial responses and 15 (48.4%) had stable disease. No new safety signals were identified. Relacorilant plus nab-paclitaxel suppressed 8 cortisol target genes of interest. The high rate of disease stabilization with relacorilant plus nab-paclitaxel in a population mostly refractory to taxanes suggests that GR modulation combined with chemotherapy should be tested in earlier mPDAC treatment settings.

## Introduction

Pancreatic cancer is the third leading cause of cancer-related death in the United States, behind lung and colon cancer. It is estimated that over 64 000 new cases of pancreatic cancer are diagnosed in the United States every year, resulting in approximately 50 000 deaths.^1^ Pancreatic cancer is recognized as a particularly lethal disease, with 1 of the lowest 5-year relative survival rates of any cancer (12%, all stages combined^1^).

The limited efficacy of chemotherapy (and other drugs) remains a major barrier to the effective treatment of pancreatic ductal adenocarcinoma (PDAC), which accounts for approximately 90% of all cases of pancreatic cancer.^2^ Glucocorticoids have been shown to reduce the efficacy of chemotherapy in preclinical models using multiple solid tumor cell lines and mouse xenograft models implanting cervical or pancreatic tumor cell lines, as well as in clinical studies of patients with solid tumors, including pancreatic and ovarian cancer.^3-6^ Activation of glucocorticoid receptors (GRs) in tumor cells inhibits chemotherapy-induced apoptosis,^3-6^ which is thought to be mediated by activation of tumor survival genes including *SGK1* and *DUSP1*.^4^ In addition, GR antagonism has been shown to reduce the expression of *SGK1* and *DUSP1* and increase sensitivity to paclitaxel-based and androgen-targeted chemotherapy.^5,7,8^ Inhibiting the effects of GR activation in tumors may therefore help to enhance sensitivity to chemotherapy and, potentially, improve patient outcomes.

Relacorilant is a small-molecule, orally administered, high-affinity, selective GR modulator (SGRM) that has resulted in enhanced chemotherapy efficacy and chemosensitivity in preclinical models of solid tumors.^5,6^ In a pancreatic cancer cell line (MIA PaCa-2), the addition of cortisol significantly inhibited paclitaxel-induced apoptosis.^5^ Relacorilant reversed this effect, partially restoring the paclitaxel-induced apoptosis.^5^ Furthermore, in xenograft models of pancreatic cancer, the combination of relacorilant plus paclitaxel significantly reduced tumor growth and delayed disease progression compared with paclitaxel alone.^6^ In the clinical setting, a phase I study (NCT02762981) found preliminary antitumor activity and durable disease control with relacorilant plus nab-paclitaxel in patients with advanced or metastatic solid tumors.^6^ Notably, changes in GR-regulated gene expression were correlated with response.^6^ Similarly, a randomized, controlled phase II study (NCT03776812) in patients with advanced, platinum-resistant ovarian cancer demonstrated that relacorilant plus nab-paclitaxel improved progression-free survival (PFS) and overall survival (OS) with minimal additional side effects, compared with nab-paclitaxel monotherapy.^9-11^ Taken together, these preclinical and clinical findings highlight a potential role for relacorilant in combination with chemotherapy in difficult-to-treat and refractory cancers, such as metastatic PDAC (mPDAC).

Here we present the results of the RELacorilant In pancreatic Adenocarcinoma with Nab-pacliTaxel (RELIANT) study, which investigated relacorilant plus nab-paclitaxel for the treatment of refractory mPDAC.

## Methods

### Study design

RELIANT (ClinicalTrials.gov identifier: NCT04329949) was an open-label, single-arm, multicenter, phase III study to assess the safety, efficacy, and pharmacokinetics (PK) of the SGRM relacorilant plus nab-paclitaxel in patients with histologically confirmed mPDAC (Supplementary Figure S1).

The study was conducted according to the ethical principles of the Declaration of Helsinki, the International Council for Harmonisation of Technical Requirements for Pharmaceuticals for Human Use—Good Clinical Practice guidelines, and all applicable local and national (US Food and Drug Administration) regulatory requirements. Approval by the institutional review board at each study site was received prior to the start of the study in accordance with an assurance filed with and approved by the US Department of Health and Human Services (Supplementary Table S1). All patients provided written informed consent prior to participation in the study.

### Patient population

Patients eligible for the study were ≥18 years old; had a histologically confirmed diagnosis of mPDAC; had received at least 2 prior lines of therapy for PDAC in any setting, including at least 1 prior gemcitabine-based therapy and at least 1 fluoropyrimidine-based therapy (prior nab-paclitaxel was not required, but was permitted); had received no more than 4 prior lines of cytotoxic or myelosuppressive therapy for PDAC; had a Karnofsky performance status (KPS) score of ≥70; and had a measurable lesion at baseline (per investigator-assessed Response Evaluation Criteria in Solid Tumors [RECIST], version 1.1).

Patients were excluded from the study if they had pancreatic neuroendocrine tumors, lymphoma of the pancreas, acinar pancreatic cancer, or ampullary cancer; had known untreated parenchymal brain metastasis or uncontrolled nervous system metastases; had received systemic corticosteroids within 21 days of study start or had a requirement for treatment with chronic or frequently used oral or inhaled corticosteroids for medical conditions or illnesses; had a rapid decline in KPS score or other factor indicative of rapid clinical deterioration, in the opinion of the investigator, prior to enrollment (during screening); or had any other condition (eg, cardiac disease and active infection) that placed them at an unacceptably high risk for toxicities, or impaired study participation or co-operation.

### Procedures and endpoints

All patients received oral relacorilant at a starting dose of 100 mg once daily, together with nab-paclitaxel (80 mg/m^2^) on days 1, 8, and 15 of each 28-day cycle. The relacorilant dose was subsequently titrated up to 150 mg once daily in 25 mg increments (at the start of treatment cycles 2 and 3), tolerability permitting. All patients, with the exception of those with an absolute neutrophil count >10 000/mm^3^, also received prophylactic granulocyte colony-stimulating factor treatment to reduce the risk of neutropenia, starting 1 day after each nab-paclitaxel infusion. At least 2 doses of filgrastim (5 μg/kg/day) were recommended, but pegfilgrastim was permitted in patients with a chemotherapy-free window of 2 weeks. Reactive granulocyte colony-stimulating factor treatment was also permitted in patients who experienced clinically meaningful neutropenia, or to maintain dose intensity. All study treatments were continued until disease progression, unacceptable toxicity, patient withdrawal, or death.

The primary efficacy endpoint was the objective response rate (ORR) as assessed by blinded independent central review (BICR) and defined as the proportion of patients who attained a confirmed complete response (CR) or partial response (PR) according to RECIST, version 1.1. Secondary efficacy endpoints included investigator-assessed ORR, duration of response (DOR; defined as the time from first CR or PR until disease progression), PFS (time from enrollment to disease progression or death), OS (time from enrollment to death), and cancer antigen 19-9 (CA19-9) response (≥50% reduction from baseline) at 8 and 16 weeks in patients who had CA19-9 greater than the upper limit of normal at baseline. The best percent change from baseline in target lesion size was also measured.

Safety was assessed from the first treatment day until 30 days after the last dose of study treatment and included the monitoring of adverse events (AEs), clinical laboratory parameters, and vital signs. AE severity was graded according to the National Cancer Institute’s Common Terminology Criteria for Adverse Events, version 5.0, and AEs were coded using the Medical Dictionary for Regulatory Activities, version 23.0.

Blood samples for pharmacodynamic assessments were collected before and 4 hours after dosing on the first day of cycle 1, before dosing on the second and last days of cycle 1 (days 2 and 15), before dosing on the first day of each subsequent cycle, and at the end-of-treatment visit. The analysis of GR-targeted gene suppression was conducted on whole blood samples using a panel of 8 genes that had been shown in a previous study to be upregulated in response to the GR agonist prednisone (*RGS2*, *DUSP1*, *MCL1*, *STAT1*, *PTGS2*, *GILZ*, *GSK3B*, and *SGK1*; data on file). To assess changes in gene expression, paired baseline and post-treatment (predose) blood samples were thawed and processed in the same batch. RNA was isolated using the PAXgene Blood RNA kit (Qiagen, Germantown, MD, USA) according to the manufacturer’s instructions, and the RNA yield was quantified using a NanoDrop ND-2000 spectrophotometer (Thermo Fisher Scientific, Waltham MA, USA). Archival or recent tumor biopsy samples were obtained during screening for the analysis of tumor GR expression levels, and optional tumor tissue was collected during the study if patient consent was provided.

Blood samples for the analysis of PK (including maximum plasma concentration [C_max_] and area under the concentration-time curve from 0 to *x* hours [AUC_0-*x*_]) were taken before and 4 hours after dosing on the first day of cycle 1, before dosing on the second day of cycle 1, and before and 0.5, 0.75, 1, 2, 4, and 6 hours after dosing on the last day of cycle 1.

### Statistical analysis

The study planned to include a total of 80 patients in the intention-to-treat (ITT) population, to provide sufficient precision to determine whether the lower bound of the 95% CI for ORR excluded 10%. In addition, a preplanned interim analysis of safety and efficacy by an independent data monitoring committee (IDMC) was planned after approximately 40 patients had been enrolled in the study and either completed 12 weeks of treatment and had at least 1 evaluable postbaseline tumor assessment, or discontinued treatment due to disease progression or toxicity. Based on the results of this interim analysis, there were 3 possible scenarios: the study could be stopped if the investigator-assessed ORR was <10%; the IDMC could consider continuing the study based on the totality of the data available if the investigator-assessed ORR was ≥10% to <20%; or the study could continue enrollment as planned if the investigator-assessed ORR was ≥20%.

All efficacy and safety analyses were performed on the ITT population, except where noted. All enrolled patients in the ITT population received at least 1 dose of study medication (relacorilant plus nab-paclitaxel). PK analyses were performed using data from all enrolled patients who had sufficient PK data available.

For the primary efficacy endpoint and CA19-9 responder analyses, point estimates and 95% CIs were calculated using the Clopper-Pearson method. For PFS, DOR, and OS, Kaplan-Meier analyses were used to determine medians, event-free rates, and 95% CIs. Sensitivity analyses were performed using the efficacy-evaluable population, defined as all patients in the ITT population who had at least 1 evaluable postbaseline radiographic tumor assessment. Safety data were summarized using standard descriptive statistics (absolute values and percentages). PK parameters were calculated using standard noncompartmental methods and summarized using descriptive statistics (geometric mean [coefficient of variation] and median [range]).

For genetic biomarker analyses, the fold change from baseline in plasma RNA was calculated using the formula:


$$\begin{array}{l} Fold\;Change=log_{2}\left(\frac{post\text{-}treatment}{baseline}\right), \end{array}$$

where the log_2_ change from baseline was calculated prior to assessing any correlation coefficients or statistical significance.

## Results

### Patients and baseline characteristics

The RELIANT study enrolled 43 patients at 17 US sites between June 2020 and May 2021. The planned interim analysis was conducted with a data cutoff date of May 25, 2021. Because the confirmed investigator-assessed ORR did not meet the predefined threshold of ≥10% at the preplanned interim analysis, enrollment was stopped on the recommendation of the IDMC.

All 43 patients received at least 1 dose of study medication (relacorilant plus nab-paclitaxel) and were included in the ITT population; 12 of 43 (27.9%) patients did not reach the first postbaseline radiographic assessment and were considered not evaluable, leaving 31 patients in the efficacy-evaluable population. Four of 43 (9.3%) patients were escalated to the highest dose of relacorilant (150 mg), and 16 of 43 (37.2%) were escalated to 125 mg.

**Figure 1.**
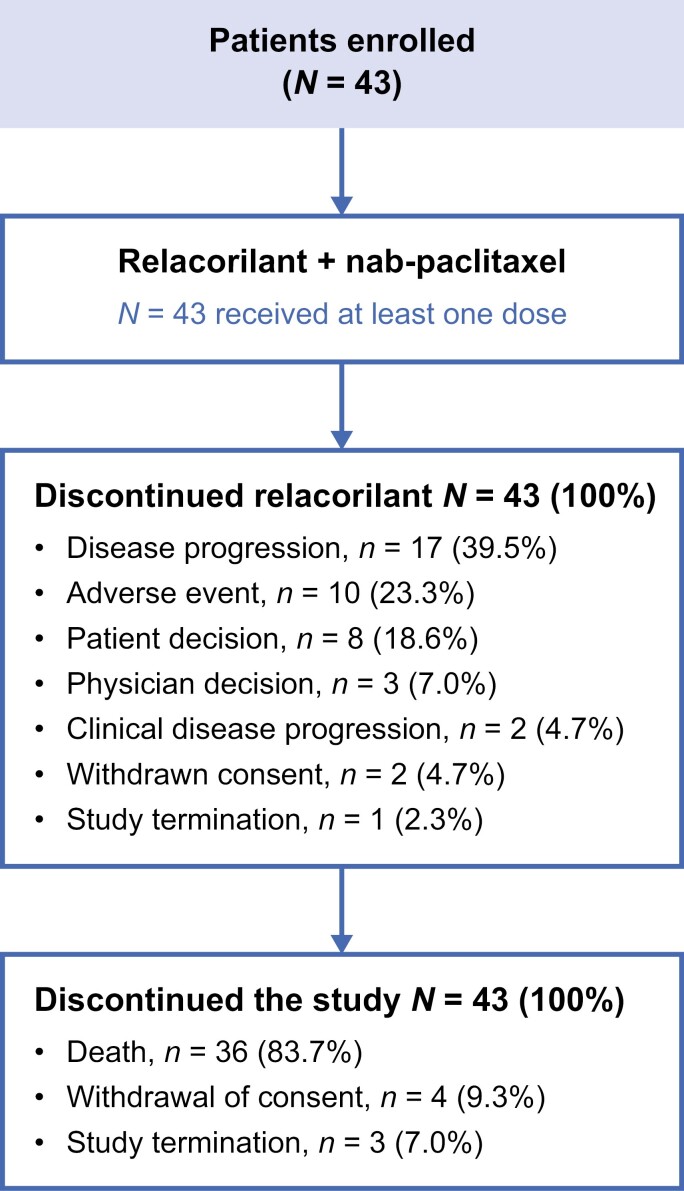
Patient disposition.

At the time of the data analysis, all patients had discontinued the study. The most common reasons for discontinuing relacorilant treatment were disease progression (17 [39.5%]), AEs (10 [23.3%]), and patient decision (8 [18.6%]; Figure 1). The median number of relacorilant treatment cycles was 1 (range, 1-15) and the median duration of treatment was 42 days (range, 5-483). For nab-paclitaxel, the median number of treatment cycles was 2 (range, 1-17) and the median duration of treatment was 36 days (range, 1-470).

Baseline patient demographics and disease characteristics are summarized in Table 1. Patients had a median age of 64 years (range, 43-78) and a median KPS score of 80 (range, 70-100); 81.4% had liver metastases and 67.4% had lung metastases. This was a heavily pretreated population in which patients had received a median of 3 prior lines of therapy in the metastatic setting, 39.5% had received 3 prior lines of therapy in any setting, and 23.3% had received 4 or more prior lines of therapy (1 patient had received 5, but this was not exclusionary). All patients had received prior gemcitabine-based and prior fluoropyrimidine-based therapy, and all but 3 patients had received prior nab-paclitaxel therapy. Of those who had not received prior nab-paclitaxel, 1 had received prior taxane therapy.

**Table 1.** Baseline patient demographics and characteristics.

Characteristic	Relacorilant + nab-paclitaxel (*N* = 43)
Age, median (range), years	64 (43-78)
Male sex, *n* (%)	24 (55.8)
Race^a^, *n* (%)	
White	35 (81.4)
Black or African American	3 (7.0)
Asian	4 (9.3)
Ethnicity, *n* (%)	
Hispanic or Latino	3 (7.0)
Not Hispanic or Latino	37 (86.0)
Not reported	3 (7.0)
KPS score at baseline, median (range)	80 (70-100)
Metastases at baseline, *n* (%)	
Liver	35 (81.4)
Lung	29 (67.4)
Number of prior lines of therapy, *n* (%)	
2	16 (37.2)
3	17 (39.5)
≥4	10 (23.3)
Number of prior therapies in the metastatic setting, median (range)	3 (2-4)
Number of prior therapies in any setting, median (range)	3 (2-5)
Prior therapy in any setting, *n* (%)	
Prior gemcitabine and prior fluoropyrimidine	43 (100)
No prior nab-paclitaxel	3 (7.0)
No prior nab-paclitaxel, but other prior taxane	1 (2.3)

^a^Race not reported for 1 patient.

Abbreviation: KPS, Karnofsky performance status.

### Efficacy

**Figure 2.**
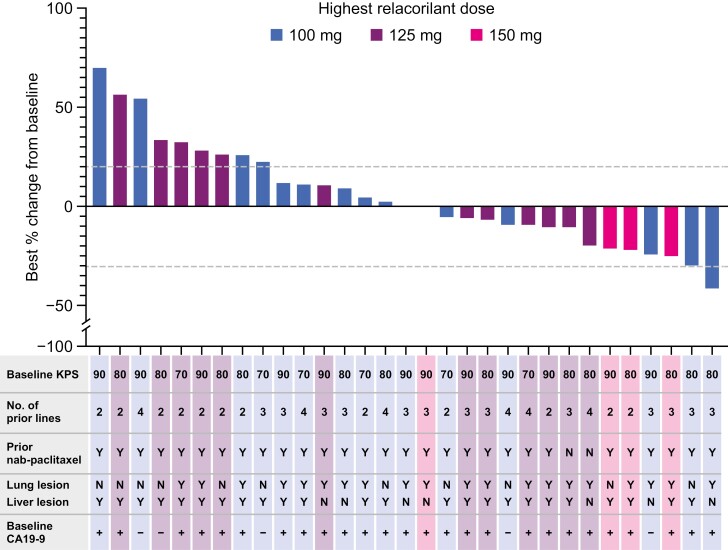
Waterfall plot showing the best percent change from baseline (as assessed by the investigator) in target lesions for all patients in the ITT population who had measurable disease at baseline and underwent a postbaseline target lesion assessment (*N* = 31). Plus signs indicate CA19-9 ≥40 U/mL; minus signs indicate CA19-9 <40 U/mL. Abbreviations: CA19-9, cancer antigen 19-9; ITT, intention-to-treat; KPS, Karnofsky performance status; N, no; Y, yes.

No patient attained a confirmed CR or PR during the study (assessed by either BICR or the investigator), resulting in an overall confirmed ORR of 0%. However, 17 patients achieved an unconfirmed investigator-assessed PR or had stable disease (2 and 15 patients, respectively; Table 2). Both patients with unconfirmed PRs received nab-paclitaxel in combination with cisplatin and gemcitabine in the neoadjuvant setting. In addition, 14 of 31 (45.2%) patients with both baseline and postbaseline target lesion assessments showed a reduction in target lesion size (best percentage change; Figure 2). Examples of 2 patients with tumor reductions are shown in Supplementary Figure S2A, S2B.

**Table 2.** Summary of disease responses (per RECIST, version 1.1)^a^.

Response	BICR-assessed response		Investigator-assessed response	
	ITT population (*N* = 43)	EE population (*n* = 31)	ITT population (*N* = 43)	EE population (*n* = 31)
ORR (confirmed), *n*	0	0	0	0
Best overall response, *n* (%)				
PR	0	0	2 (4.7)	2 (6.5)
SD	21 (48.8)	20 (64.5)	15 (34.9)	15 (48.4)
PD	11 (25.6)	10 (32.3)	14 (32.6)	14 (45.2)
Nonevaluable	11 (25.6)	1 (3.2)	12 (27.9)	0

^a^Duration of response is not applicable because ORR = 0%.

Abbreviations: BICR, blinded independent central review; EE, efficacy-evaluable; ITT, intention-to-treat; ORR, objective response rate; PD, progressive disease; PR, partial response; RECIST, Response Evaluation Criteria in Solid Tumors; SD, stable disease.

After a median follow-up of 5.8 months, the BICR-assessed median PFS was 2.4 months (95% CI, 1.4-4.2), the investigator-assessed median PFS was 1.6 months (95% CI, 1.4-3.1), and the median OS was 3.9 months (95% CI, 2.8-4.9) in the ITT population.

At baseline, 39 of 43 (90.7%) patients had elevated CA19-9 levels. Following 8 weeks of treatment with relacorilant plus nab-paclitaxel, CA19-9 levels had decreased in 17 (43.6%) of these patients; in 5 (12.8%) patients this decrease was ≥50%, and in 7 (17.9%) patients it was ≥20%.

### Safety

All patients experienced at least 1 treatment-emergent AE of any grade, most commonly fatigue, nausea, decreased appetite, anemia, and vomiting (Table 3). The most common AEs related to relacorilant were fatigue (24 [55.8%]), decreased appetite (12 [27.9%]), and nausea (12 [27.9%]), and the most common AEs related to nab-paclitaxel were fatigue (27 [62.8%]), nausea (16 [37.2%]), decreased appetite (12 [27.9%]), neutrophil count decreased (12 [27.9%]), and vomiting (12 [27.9%]).

**Table 3.** Treatment-emergent AEs occurring in ≥20% of patients.

*n* (%)	Any-grade AE (*N* = 43)	Grade ≥3 AE (*N* = 43)
Any AE	43 (100)	37 (86.0)
Fatigue	35 (81.4)	13 (30.2)
Nausea	21 (48.8)	1 (2.3)
Decreased appetite	17 (39.5)	1 (2.3)
Vomiting	15 (34.9)	1 (2.3)
Anemia	14 (32.6)	9 (20.9)
Hypoalbuminemia	12 (27.9)	1 (2.3)
Neutrophil count decreased	12 (27.9)	5 (11.6)
Abdominal pain	11 (25.6)	4 (9.3)
Back pain	11 (25.6)	1 (2.3)
Diarrhea	10 (23.3)	1 (2.3)
Hypokalemia	10 (23.3)	5 (11.6)
Hyponatremia	9 (20.9)	2 (4.7)
Peripheral edema	9 (20.9)	0
White blood cell count decreased	9 (20.9)	8 (18.6)

Abbreviation: AE, adverse event.

The majority of patients (37 [86.0%]) also experienced at least 1 grade ≥3 AE, most commonly fatigue (13 [30.2%]), anemia (9 [20.9%]), and decreased white blood cell count (8 [18.6%]).

Serious AEs deemed to be related to relacorilant treatment occurred in 14.0% of patients (10 events in 6 patients), and included fever, fatigue, febrile neutropenia, pneumonitis, failure to thrive, diarrhea, vomiting, pancytopenia, abdominal pain, and ascites (1 each [2.3%]). Most patients (32 [74.4%]) did not require relacorilant dose reductions or discontinuations as a result of AEs; 10 (23.3%) patients had dose discontinuations, 1 (2.3%) patient had a dose reduction, and 28 (65.1%) patients had temporary dose interruptions, mostly during periods when the nab-paclitaxel dose was also interrupted. In addition, AEs led to nab-paclitaxel dose discontinuations in 9 (20.9%) patients, dose reductions in 13 (30.2%) patients, and temporary dose interruptions in 20 (46.5%) patients.

Three (7.0%) patients died as a result of AEs: 1 each due to cardiorespiratory arrest, pneumonitis, and pulmonary embolism. Only the death due to pneumonitis was considered by the investigator to be treatment-related (specifically related to both relacorilant and nab-paclitaxel).

### Pharmacokinetics

Primary PK parameters for relacorilant 100 mg daily and nab-paclitaxel following continuous daily dosing (cycle 1, day 15) are summarized in Supplementary Table S2. When administered concurrently, the mean maximum concentrations and exposures for relacorilant and nab-paclitaxel were 375 and 2380 ng/mL (C_max_), and 3400 and 2560 ng·h/mL (AUC_0-24_ and AUC_0-6_), respectively. Overall, the range of nab-paclitaxel exposures was consistent with those published for nab-paclitaxel 100-125 mg/m^2^ monotherapy.^11,12^

### Pharmacodynamics

Tumor GR expression was detected in all 11 evaluable biopsy samples, although GR expression levels were independent of clinical outcome (ie, no significant difference in expression between patients who had OS/PFS above or below the median, or between those with a best response of CR/PR versus stable/progressive/nonevaluable disease).

**Figure 3.**
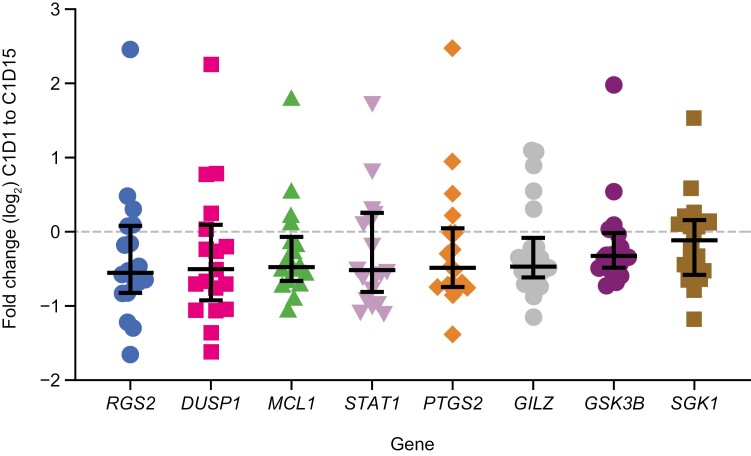
Change in expression of 8 genes following relacorilant plus nab-paclitaxel treatment on whole blood RNA analysis. The genes are activated by cortisol and known to be upregulated in response to a GR agonist (prednisone). Abbreviations: C, cycle; D, day; GR, glucocorticoid receptor.

Based on whole blood RNA analysis (*n* = 21 paired samples), the expression of 8 cortisol target genes of interest (*RGS2*, *DUSP1*, *MCL1*, *STAT1*, *PTGS2*, *GILZ*, *GSK3B*, and *SGK1*) known to be upregulated in response to a GR agonist (prednisone; data on file) was suppressed by treatment with relacorilant plus nab-paclitaxel (Figure 3). No significant effect of relacorilant was observed with any other biomarkers assessed, including 11-deoxycortisol levels and homeostatic model-assessed insulin resistance.

## Discussion

Major barriers to the effective treatment of PDAC include the limited efficacy of chemotherapy and the paucity of targetable molecular alterations.^2^ Activation of GRs on tumor cells results in increased expression of tumor survival genes such as *SGK1* and *DUSP1*, thereby decreasing the efficacy of chemotherapy.^4-8^ This has led to the development of treatments aimed at the modulation of GR signaling (ie, SGRMs), to enhance the efficacy of cytotoxic agents.^5,6^

Results from this open-label study (RELIANT) investigating the efficacy and safety of the SGRM relacorilant in combination with nab-paclitaxel for the treatment of patients with mPDAC provide support for this treatment approach; this is despite the study being terminated by the sponsor due to the interim analysis not meeting the robust prespecified efficacy requirements (≥10% ORR in a heavily pretreated population). Of the 31 efficacy-evaluable patients, 2 (6.5%) attained an unconfirmed PR (as assessed by the investigator), and nearly half (15 [48.4%]) had stable disease, 1 of whom was still alive at the end of the study after more than 1 year of treatment. In addition, reductions in target lesion size were observed in close to half of all efficacy-evaluable patients (45.2%), and nearly half (43.6%) of patients with elevated baseline levels showed a reduction in the tumor marker CA19-9 after 8 weeks of treatment. Patients were heavily pretreated and had poor prognostic features. Nearly two-thirds (62.8%) of patients had received 3 or more prior lines of therapy. Because of this, RELIANT may be considered a demonstration of moderate efficacy, considering that historical response rates are typically very low (~0%) in third-line mPDAC, as are PFS and OS (<6 months), even with standard combination therapy.^13,14^ In RELIANT, all but 3 patients had received prior nab-paclitaxel treatment. Effective third-line and later line treatment options for PDAC are limited^2,15,16^; therefore, disease stabilization with the addition of relacorilant to nab-paclitaxel in a population with mostly nab-paclitaxel–refractory mPDAC is notable. It is possible that the addition of relacorilant to standard-of-care therapy in earlier treatment settings may result in more pronounced efficacy.

The overall safety profile of relacorilant plus nab-paclitaxel in the current study was consistent with previous studies in patients with cancer^6,9^ with no toxicities other than those already associated with nab-paclitaxel (eg, neutropenia). While some serious AEs were assessed to be associated with relacorilant treatment, all but 1 resolved, and the cases were likely confounded by both the concomitant nab-paclitaxel treatment and the severity of the underlying disease.

PK parameters for relacorilant plus nab-paclitaxel were consistent with those published previously.^6^ In particular, when administered in combination with relacorilant, the exposure of nab-paclitaxel at a dose of 80 mg/m^2^ was similar to monotherapy doses of 100-125 mg/m^2^ (125 mg/m^2^ is the approved monotherapy dose for patients with PDAC^6^). The greater nab-paclitaxel exposure in the combination therapy regimen is likely due to relacorilant being a strong inhibitor of CYP3A4, whereas nab-paclitaxel is metabolized by CYP3A4 (as well as CYP2C8^17^). These PK data from the current study further validate the recommended phase II dose for relacorilant plus nab-paclitaxel.^6^

Whole blood RNA analysis in our study confirmed that the combination of relacorilant plus nab-paclitaxel suppressed expression of all 8 cortisol target genes tested (*RGS2*, *DUSP1*, *MCL1*, *STAT1*, *PTGS2*, *GILZ*, *GSK3B*, and *SGK1*), indicating that the doses of relacorilant used in the study were sufficient to achieve GR modulation. It would be interesting in future studies to assess whether cortisol target genes are associated with patient outcomes in different tumor types. A previous phase I study of relacorilant plus nab-paclitaxel in patients with heavily pretreated solid tumors (75% refractory to taxane therapy) showed clinical benefits that were associated with GR-regulated transcript-level changes in a panel of GR-controlled genes, in particular, *CD163*, *IGF2R*, and genes encoding immunomodulatory drug targets such as *CXCL8*, *PTGER4*, and *IDOL*.^6^

There are several limitations to the study that should be considered when interpreting the results. First, it was terminated early after the ORR did not meet the prespecified cutoff at the interim analysis. Thus, the results presented are limited to the 43 patients included in the interim analysis, which restricts the interpretation of the observed efficacy. Secondly, this was an open-label study in a heavily pretreated patient population. Due to the lack of a comparator arm, clear attribution of any AEs to either relacorilant or nab-paclitaxel was not possible. Moreover, the lack of significant efficacy hinders translational correlative work with baseline tumor GR expression and pharmacodynamic effects. Lastly, tumor tissue was not available for GR-targeted gene expression analyses, so these analyses were performed using whole blood.

In conclusion, while relacorilant plus nab-paclitaxel showed modest antitumor activity in patients with mPDAC, most of whom were nab-paclitaxel–refractory and heavily pretreated, the level of benefit did not justify further study as a treatment for end-stage pancreatic cancer. The modest antitumor activity seen may warrant further exploration in earlier lines of therapy or in another clinical situation such as maintenance therapy for mPDAC (this would be justified if prolonged disease stabilization is observed). Relacorilant plus nab-paclitaxel continues to be evaluated for the treatment of other tumor types, including ovarian cancer (NCT05257408).

## Supplementary Material

Supplementary material is available at *The Oncologist* online.

oyae210_suppl_Supplementary_Material

## Data Availability

Additional data are provided in the manuscript supplement available online. De-identified datasets for the results reported in this publication may be made available to qualified researchers following the submission of a methodologically sound proposal to datarequests@corcept.com. Data will be made available for such requests following the online publication of this article and for 1 year thereafter in compliance with applicable privacy laws, data protection, and requirements for consent and anonymization. Data will be provided by Corcept Therapeutics.
