# Supplementary material for: Relacorilant plus nab-paclitaxel for the treatment of metastatic pancreatic ductal adenocarcinoma: results from the open-label RELIANT study
Source: Oncologist. 2024 Aug 26;29(11):957–65. doi: 10.1093/oncolo/oyae210 (PMC11546724; doi:10.1093/oncolo/oyae210)
Supplement: oyae210_suppl_Supplementary_Material [file oyae210_suppl_supplementary_material.pdf]

SUPPLEMENTARY MATERIAL

Relacorilant Plus Nab-Paclitaxel for the Treatment of Metastatic Pancreatic Ductal Adenocarcinoma: Results From the Open-Label RELIANT Study

Erkut H. Borazanci, Nathan Bahary, Vincent Chung, Timothy K. Huyck, Ebenezer A. Kio, Elena Gabriela Chiorean, Roland T. Skeel, Olatunji B. Alese, Dana B. Cardin, Christos Fountzilas, Wahid T. Hanna, Alexis D. Leal, Valerie Lee, Anne M. Noonan, Philip A. Philip, Zev A. Wainberg, Hristina Pashova, Grace Mann, Paul E. Oberstein

Table of Contents

Supplementary Table S1. Institutional Review Boards. 2

Supplementary Table S2. Primary PK parameters for relacorilant plus nab-paclitaxel after continuous dosing (cycle 1, day 15). 3

Supplementary Figure S1. Study design. 4

Supplementary Figure S2. Examples of tumor reduction (CT and FDG-PET scans) after treatment with relacorilant plus nab-paclitaxel. 5

Supplementary Table S1. Institutional Review Boards.

Institutional Review Board Roswell Park Cancer Institute	UCLA Office of the Human Research Protection Program (OHRPP)
John Hopkins University Institutional Review Board	Vanderbilt University Institutional Review Board
NYU School of Medicine Institutional Review Board	Western Institutional Review Board
The University of Toledo – Human Research Protection Program – Cancer Biomedical Institutional Review Board	

Supplementary Table S2. Primary PK parameters for relacorilant plus nab-paclitaxel after continuous dosing (cycle 1, day 15).

	**Relacorilant (100 mg)**		**Nab-paclitaxel (80 mg/m^2)**	
**Statistical parameter**	**C_{max} (ng/mL)**	**AUC_{0-24} (ng·h/mL)**	**C_{max} (ng/mL)**	**AUC_{0-6} (ng·h/mL)**
	n = 24	*n* = 18	*n* = 26	*n* = 19
Geometric mean (CV%)	375 (89)	3400 (108)	2380 (78)	2560 (60)
Median (range)	404 (91-1150)	2920 (899-16900)	2300 (551-8160)	2880 (1020-6340)

Abbreviations: AUC_{0-24}, area under the concentration-time curve from 0 to 24 hours; AUC_{0-6}, area under the concentration-time curve from 0 to 6 hours; C_{max}, maximum plasma concentration; CV%, coefficient of variation; PK pharmacokinetic.

Supplementary Figure S1. Study design.

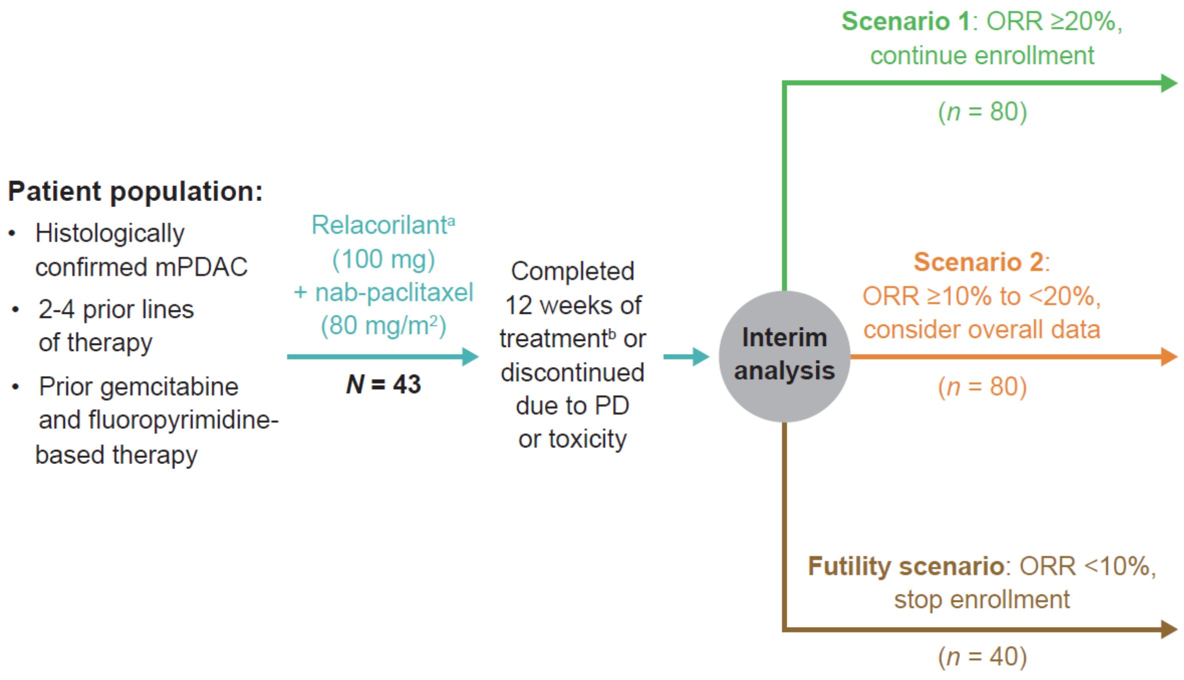

[a]Starting 100 mg once daily with titration up to 150 mg once daily in 25 mg increments.

[b]Including second radiographic assessment and at least one postbaseline tumor assessment other than nonevaluable.

Abbreviations: mPDAC, metastatic pancreatic ductal adenocarcinoma; ORR, objective response rate; PD, progressive disease.

Supplementary Figure S2. Examples of tumor reduction (CT and FDG-PET scans) after treatment with relacorilant plus nab-paclitaxel.

A

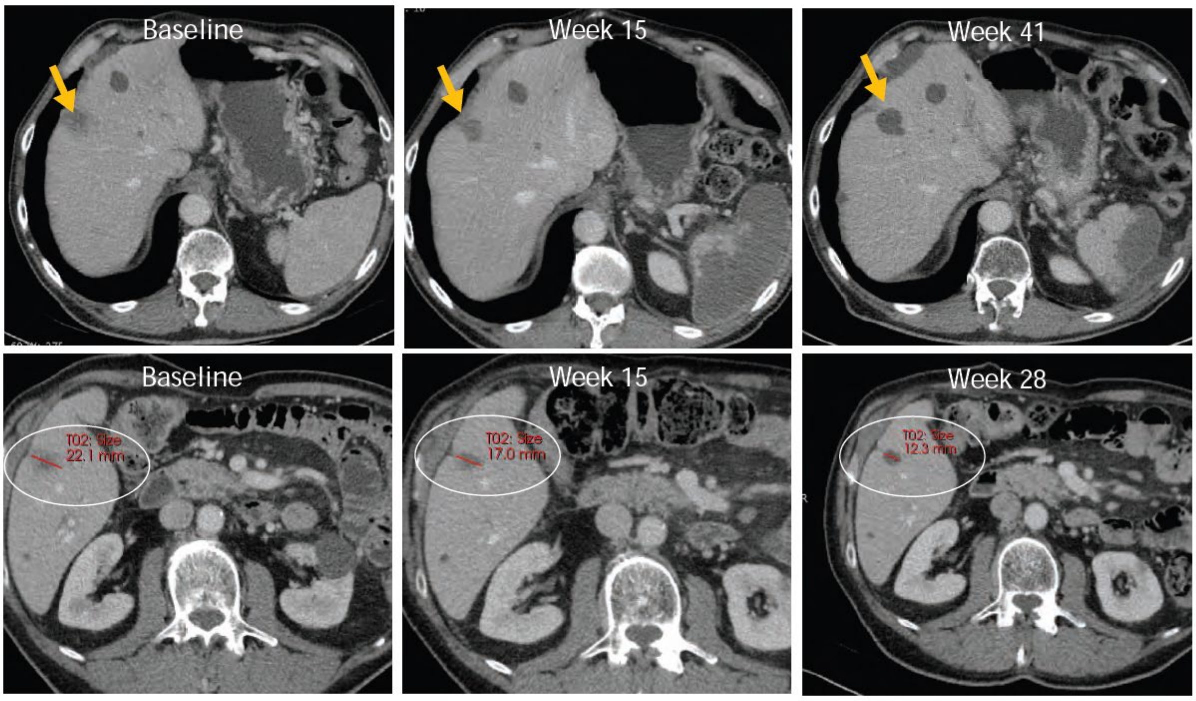

Images from a 73-year-old male patient who had received three prior lines of therapy, including nab-paclitaxel at baseline. The patient then received nine cycles of relacorilant plus nab-paclitaxel treatment. Multiple heterogeneously appearing hepatic metastases were present at baseline (yellow arrow and white circle). During treatment, the metastatic lesions demonstrated tumor shrinkage and necrosis over time. Two target lesions are shown (one in each row). At week 8, the patient's CA19-9 levels had decreased by 93.1%, from 16,275 U/mL to 1115 U/mL.

B

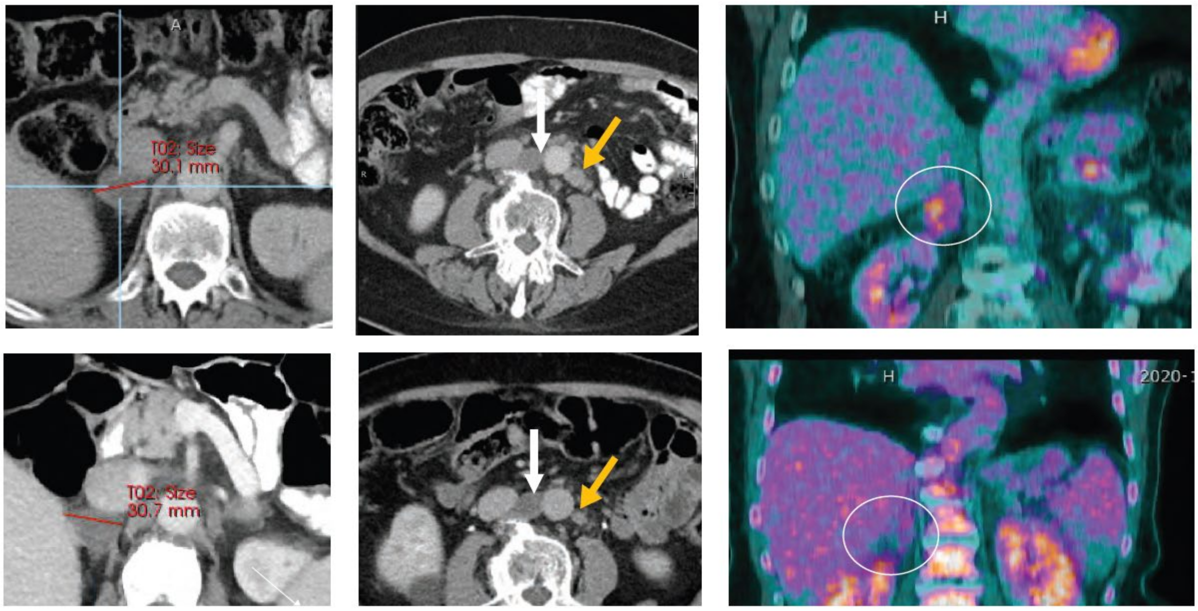

Images from a 74-year-old male patient who had received three prior lines of therapy, including nab-paclitaxel. The patient then received 17 cycles of relacorilant plus nab-paclitaxel treatment. Metastatic disease, with FDG-PET avid uptake involving the right adrenal gland and several retroperitoneal lymphadenopathies, was noted at baseline (top row). At week 7 (bottom row), improvements were observed in the FDG-PET images, with retroperitoneal lymph node stability and shrinkage (yellow arrow indicates left periaortic lymph node; white arrow indicates aortocaval lymph node; white circle indicates right adrenal gland). At week 8, the patient's CA19-9 levels had decreased by 17.6%, from 37.4 U/mL to 30.8 U/mL.

Abbreviations: CA19-9, cancer antigen 19-9; CT, computed tomography; FDG-PET, fluorodeoxyglucose positron emission tomography.
